# Inhaled turmerones can be incorporated in the organs via pathways different from oral administration and can affect weight-gain of mice

**DOI:** 10.1038/s41598-022-15168-9

**Published:** 2022-06-30

**Authors:** Yuki Takemoto, Chihiro Kishi, Hinano Ehira, Nobutaka Matsui, Taichi Yamaguchi, Yuri Yoshioka, Shinichi Matsumura, Tatsuya Moriyama, Nobuhiro Zaima

**Affiliations:** 1 Department of Applied Biological Chemistry, Graduate School of Agriculture, Kindai University, 204-3327 Nakamachi, Nara City, Nara, 631-8505 Japan; 2 INABATA KORYO, Co., Ltd, 3-5-20 Tagawa, Yodogawa, Osaka, 532-0027 Japan; 3 Agricultural Technology and Innovation Research Institute, Kindai University, Nara, 631-8505 Japan

**Keywords:** Mass spectrometry, Lipids

## Abstract

Turmerones (α-turmerone, β-turmerone, and ar-turmerone) are the major volatile compounds in turmeric (*Curcuma longa*), a perennial herb of the ginger family. We previously reported that inhaled volatile turmerones could be transferred in the blood and organs. However, the difference between the two pathways, oral administration and inhalation, and the effect of inhaled turmerones on biological activities remain unknown. In this study, we compared the distribution patterns of turmerones after oral administration and inhalation. The relative levels (concentrations of turmerones in each organ/serum) in the lung, olfactory bulb, brain, heart, kidney, and epididymal fat in the inhalation group tended to be, or are significantly, higher than in the oral administration group. The relative levels of brown adipose tissue in the inhalation group were lower than in the oral administration group. Long-term (50 days) inhalation to volatile turmerones suppressed weight gain and hypertrophy of adipocytes in the epididymal fat of mice fed a high-fat diet. These results suggest that inhaled turmerones can be incorporated into the organs of mice via different pathway from as to those from oral administration and can affect the biological function of the organs under certain conditions.

## Introduction

Turmeric (Curcuma longa) is a perennial herb of the ginger family and is used as a coloring agent, spice, or flavoring agent. Curcumin is well-known as the main active component of turmeric. However, many chemical substances have been identified in turmeric, and various medicinal effects have been reported in curcumin-free turmeric extracts, suggesting the existence of bioactive components other than curcumin^1^.

Turmerones (α-turmerone, β-turmerone, and ar-turmerone) are characteristic compounds in turmeric^2^. In vitro studies have reported effects on several biological activities, including anti-inflammatory^3–5^, anti-immunomodulatory^6^, anti-proliferative activity for cultured cancer cell^7,8^, and antifungal activities^9^, and inhibitory effects on β-secretase^10^. Moreover, turmerones have demonstrated several in vivo biological activities such as anti-depressant^11^, anti-convulsant^12^, blood glucose-lowering^13,14^, and antioxidant effects^15^, as well as differentiation-inducing effects on neural stem cells^16^. Oral administered turmerones are reportedly distributed in their intact form in the organs of mice^17^. These data suggest that turmerones are promising candidates as functional food factors that can prevent some diseases.

In addition to the above-mentioned function, volatility is another property of turmerones. Volatile turmerones are one of main components of flavor of turmeric or turmeric oil. Recently, we found that inhaled volatile turmerones can be transferred into the blood and organs of mice^18^, indicating that bioactive turmerones have two different pathways into the body. However, the difference between the two pathways, oral administration and inhalation, and the effects of inhaled turmerones on biological activities remain unknown. In this study, we estimated the distribution property of volatile turmerones and the possible effects on biological activities.

## Results

### Evaluation of turmerones in turmeric oil and volatile turmerones in a flask

**Figure 1:**
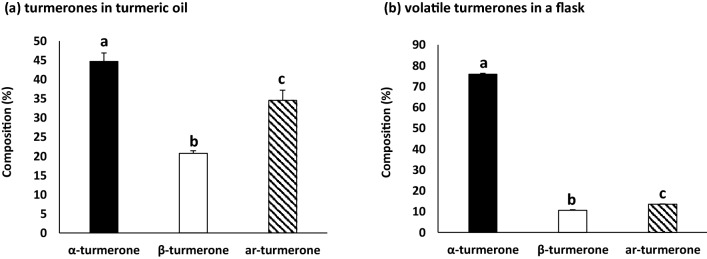
Composition of turmerones in turmeric oil and volatile turmerones in a flask. (**a**) Composition of turmerones in turmeric oil (n = 3). (**b**) Composition of volatile turmerones in a flask (n = 6). The values are expressed as the mean ± standard deviation. Values with different letters are significantly different among turmerones (*p* < 0.05).

The composition of turmerones in turmeric oil was 44.72 ± 2.14% α-turmerone, 20.77 ± 0.63% β-turmerone, and 34.51 ± 2.69% ar-turmerone (Fig. 1a). The composition of volatile turmerones in a flask was 75.87 ± 0.36% α-turmerone, 10.62 ± 0.18% β-turmerone, and 13.51 ± 0.19% ar-turmerone (Fig. 1b).

### Comparison of transfer ratio of turmerones between oral administration and inhalation

**Figure 2:**
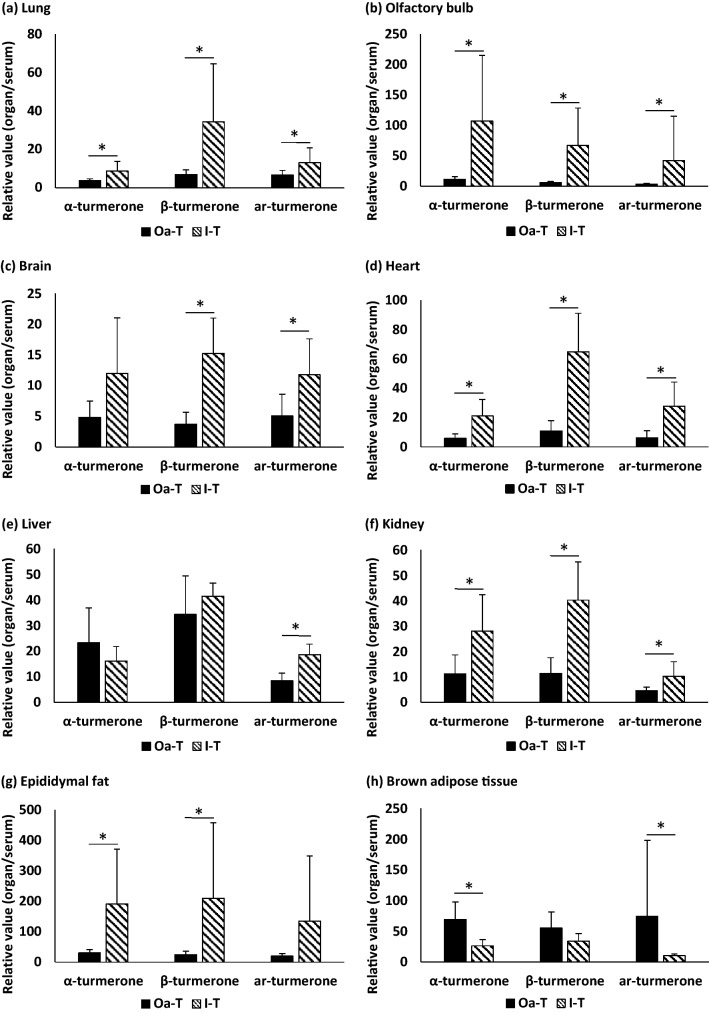
Comparison of turmerones distribution between Oa-T and I-T groups. (**a**) Lung. (**b**) Olfactory bulb. (**c**) Brain. (**d**) Heart. (**e**) Liver. (**f**) Kidney. (**g**) Epididymal fat. (**h**) Brown adipose tissue. Oa-T group, no inhalation + turmeric solution (0.04 mg/g) (n = 5); and I-T group, volatile turmeric oil inhalation (60 min) (n = 6). Composition values are calculated using the equation. Amount of each turmerone in each organ/amount of each turmerone in each serum. The values are expressed as the mean ± standard deviation. * means significant difference between Oa-T group and I-T group (*p* < 0.05).

The different composition between samples used for oral administration and inhalation made it difficult to compare distribution property. Therefore, we focused on comparison of transfer ratio of turmerones from blood to the organs between oral administration and inhalation. Transfer ratio (relative values: concentration of turmerones in each organ/concentration of turmerones in serum) in each organ is shown in Fig. 2. In the lung, olfactory bulb, heart, and kidney, the relative levels of all turmerones were significantly higher in the I-T group than in the Oa-T group. In the brain, the relative level of β-turmerone and ar-turmerone were significantly higher in the I-T group than in the Oa-T group. The level of α-turmerone tended to be higher in the I-T group than in the Oa-T group. In the epididymal fat, the relative level of α-turmerone and β-turmerone were significantly higher in the I-T group than in the Oa-T group. The level of ar-turmerone tended to be higher in the I-T group than in the Oa-T group. In the brown adipose tissue, the relative level of α-turmerone and ar-turmerone were significantly lower in the I-T group than in the Oa-T group. The level of β-turmerone tended to be lower in the I-T group than in the Oa-T group. In the liver, the relative level of ar-turmerone was significantly higher in the I-T group than in the Oa-T group. The levels of α-turmerone tended to be lower in the I-T group than in the Oa-T group. There was no significant difference between the level of β-turmerone in the I-T and Oa-T groups.

### Evaluation of turmerones inhalation on body weight and serum parameters

**Figure 3:**
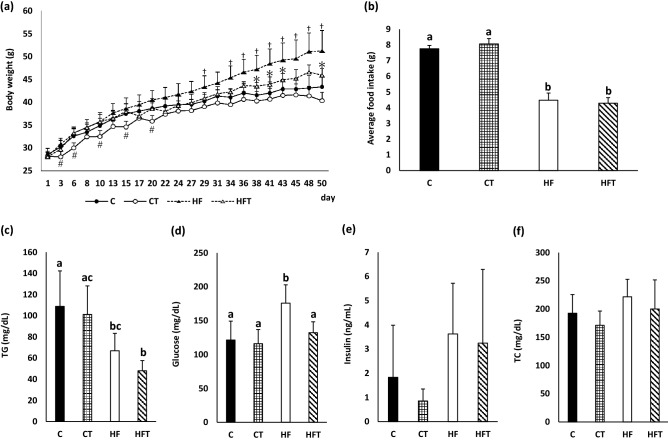
Body weight change, food intake and serum parameters. (**a**) Body weight change. # means significant difference between C group and CT group (*p* < 0.05). † means significant difference between C group and HF group (*p* < 0.05). * means significant difference between HF group and HFT group (*p* < 0.05). (**b**) Average food intake. (**c**) Serum triglyceride (TG) levels. (**d**) Serum glucose levels. (**e**) Serum insulin levels. (**f**) Serum total cholesterol (TC) levels. The C group, control diet + no inhalation (n = 8); the CT group, control diet + volatile turmeric oil inhalation (60 min) (n = 7); the HF group, high-fat diet + no inhalation (n = 5); and the HFT group, high-fat diet + volatile turmeric oil inhalation (60 min) (n = 5). The values are expressed as the mean ± standard deviation. Values with different letters are significantly different among four groups (*p* < 0.05).

We next estimated the effects of turmerones inhalation on the body weight and serum parameters of mice fed a normal or high-fat diet. Weight changes were evaluated for 50 days (Fig. 3a). On days 3, 6, 10, 15, and 20, body weight in the CT group (control diet + volatile turmeric oil inhalation (60 min)) was significantly lower than that in the C group (control diet + no inhalation). However, there were no significant differences in the final body weights between the groups. On days 29 and 34–50, body weights in the HF group (high-fat diet + no inhalation) were significantly higher than those in the C group. On days 38–43 and 50, body weights in the HFT group (high-fat diet + volatile turmeric oil inhalation (60 min)) were significantly lower than those in the HF group. Turmerone did not affect the average food intake (Fig. 3b). Serum triglyceride levels in the high-fat diet groups (HF and HFT groups) were significantly lower than those in the control diet groups (C and CT groups; Fig. 3c). Blood glucose levels in the HF group were significantly higher than those in the C group. Blood glucose levels in the HFT group were significantly lower than those in the HF group (Fig. 3d). There were no significant differences in insulin and total cholesterol levels among the four groups (Fig. 3e and f).

### Evaluation of turmerones inhalation on epididymal fat

**Figure 4:**
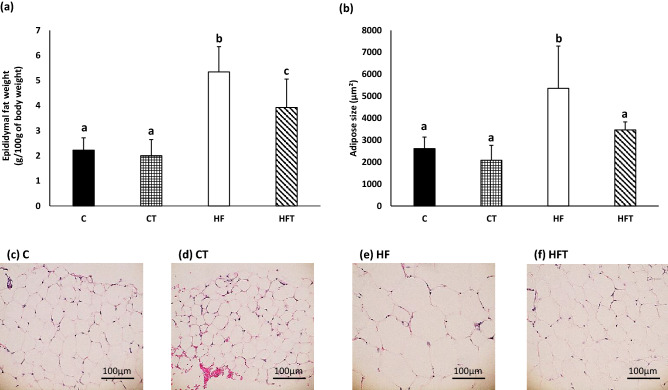
Histopathology of epididymal fat. (**a**) Epididymal fat weight. (**b**) Adipocyte size (μm^2^). (**c**–**f**) Representative images of HE staining. The C group, control diet + no inhalation (n = 8); the CT group, control diet + volatile turmeric oil inhalation (60 min) (n = 7); the HF group, high-fat diet + no inhalation (n = 5); and the HFT group, high-fat diet + volatile turmeric oil inhalation (60 min) (n = 5). The values are expressed as the mean ± standard deviation. Values with different letters are significantly different among four groups (*p* < 0.05).

Because decreased body weight was observed in the HFT group, we next investigated epididymal fat. The epididymal fat weight in the HF group was significantly higher than that in the C and CT groups, whereas in the HFT group, it was significantly lower than that in the HF group (Fig. 4a). The adipocyte size in the HF group was significantly larger than that in the C and CT groups, whereas in the HFT group, it was significantly smaller than that in the HF group (Fig. 4b–f).

## Discussion

In this study, we found a different distribution pattern of turmerones between the oral administration and inhalation routes. Based on the distribution tendency, the distribution pattern can be roughly classified into three groups: Group 1 (inhalation ≧ oral administration); the lung, olfactory bulb, brain, heart, kidney, and epididymal fat were categorized in this group; Group 2 (inhalation ≦ oral administration): the brown adipose tissue was categorized in this group; Group 3 (others): the liver was categorized in this group. The reason for the difference between Group 1 and Group 2 can be partly explained by the location of each organ. Inhaled turmerones are first transferred into the lung. Turmerones can be incorporated into the blood via the lung and it is possible that the rest of turmerones leak into the body from the lung. The higher relative levels of turmerones in the lung, heart, kidney, and epididymal fat of the I-T group can be attributed to the leakage route. Brown adipose tissue is spatially isolated from other organs, which can explain the lower distribution of all turmerones in the I-T group. It is speculated that volatile turmerones can be distributed directly into the olfactory bulb and brain through the nasal passages. Kumar et al. reported the possibility of the distribution of nasally administered pharmaceuticals to both the cerebrospinal fluid and blood^20^. Direct distribution through the nasal cavity may be a potential pathway to the brain. However, the relative size of the olfactory bulb compared to the rest of the brain is very small in primates including humans, composing about 0.01% of the human brain by volume compared to 2% of the mouse brain^21^. Whether the same can happen in humans is a topic of future research.

The above-mentioned hypothesis can partly explain the difference between oral administration and inhalation; however, our results suggest the existence of unknown mechanisms. For example, different relative levels among turmerones were observed in the liver, which may be associated with xenobiotic metabolism in the liver. In addition, distributions of α-turmerone in the brain and ar-turmerone in the epididymal fat, and β-turmerone in the brown adipose tissue cannot be categorized into above-mentioned groups, suggesting the existence of molecular structure specific distribution.

Inhalation of turmerones suppressed an increase in weight gain and serum glucose in mice fed a high-fat diet. It has been reported that ar-turmerone in turmeric extract stimulates the differentiation of human adipocytes in vitro and has ligand-binding activity for PPARγ^13,14^. An ethanol extract of turmeric reportedly suppresses an increase in blood glucose in type 2 diabetic KK-Ay mice^13,14^. Because previous data were obtained from oral administration, it should be needed to confirm whether same can occur in mice which were inhaled volatile turmerones. Body weight in the CT group was significantly decreased at early stage compared with that in the C group. At final body weight, there were no significant differences in mice fed a normal diet. These data suggest that unidentified conditions are associated with the effects of inhaled turmerones on glucose and lipid metabolism. Peripheral nerves are reportedly associated with energy metabolism in adipose tissue^22^. Observed effects in this study might be involved in nerve systems.

In conclusion, this study showed different distribution pathways between oral administration and inhalation and indicated the potential effects of inhaled turmerones on biological activities. Our findings suggest the possibility that volatile aroma components, including turmerones, can affect the living body under certain conditions. Limitation of this study needs to be noted. Mechanisms underlying the decreased body weight in CT group at early stage remain unknown. Because of the difficulty to prepare isolated turmerones, a mixture of turmerones was used in this study, which made it complicate to investigate the mechanisms of in vivo bioactivities of turmerones. It is not clear if the all turmerones act in a similar way and if active metabolites are produced. Further studies, including potential toxicity, are needed.

## Materials and methods

### Materials

Commercially available turmeric oil from the rhizomes of *C. longa* Linne was obtained from Arjuna Natural Pvt., Ltd., Alwaye, India). The collection of the rhizomes of *C. longa* Linne comply with relevant institutional, national, and international guidelines and legislation. Fresh rhizomes of *C. longa* Linne, the cultivated variety IISR Alleppey Supreme, were collected from a farm in Alleppey, Kerala, India, and were dried according to the method proposed by Jayashree et al.^23^. Permission was obtained from the land owner by Arjuna Natural PVT., Ltd. The rhizomes were authenticated by a qualified botanist at Arjuna Natural Pvt. Ltd. (ID: AE-HBRS-22). Cleaned rhizomes were fed into a back-loading type of mini pulverizer (PILOT 612; Pilotsmith India Pvt. Ltd., Kerala, India). The disintegrated material from the outlet was collected and sieved through a 14–16 mesh screen. Crude turmeric oil (310 g) was obtained from the milled rhizome (10 kg) using steam distillation in a Clevenger-type apparatus (Magnum Glass Works, Kerala, India). Crude turmeric oil (100 g) was distilled under low pressure (boiling point 120 ℃ at 150 Pa) to remove compounds with low boiling points. The oil was further distilled at > 150 ℃ to obtain a turmerone-rich fraction (74 g) of turmeric oil. Turmeric oil was stored at 4 ℃ in a brown-glass bottle for a maximum of six weeks. Turmerones used as samples for quantitation were purified from rhizomes using an n-hexane extract as follows: the extract was subjected to preparative HPLC (LC-8A system; Shimadzu Co., Ltd., Kyoto, Japan) with the following conditions: column (L-Column octadecylsiyl; length: 250 mm; particle size: 5 μm; inner diameter (I.D.): 20 mm; Chemicals Evaluation and Research Institute, Tokyo, Japan); mobile phase: water:acetonitrile (20:80, v/v); flow rate: 18.9 mL/min; detection: UV 190 and 243 nm; retention times: 12.5 min for α-turmerone, 11.4 min for β-turmerone, and 8.4 min for ar-turmerone.

### Evaluation of turmerones in turmeric oil and volatile turmerones in a flask

The turmerone concentrations in turmeric oil and in a flask were measured using a gas chromatography-mass spectrometer (Agilent 6890A-5973 N; Agilent Technologies, Santa Clara, CA, USA). The quantification conditions for turmerones in a flask has been described in a previous study^18^. The turmerone concentrations in a flask were determined as follows; Turmeric oil (10 mL) was soaked in absorbent cotton and placed in a net (mesh: 0.18 cm^2^, Hatto Co., Wakayama, Japan). It was hung using a paper string (Kouyu Co., Ltd., Fukushima, Japan) and the flask was closed with a silicone stopper. After 60 min, turmerones were adsorbed using a Tenax TA cartridge (length: 17.8 cm, outer diameter: 6 mm, I.D.: 4 mm, Sigma Aldrich Co., St. Louis, MO, USA) at 3 cm from the bottom of the flask to sample 100 mL of air.

### Animals

All animal experiments were approved by the Animal Care and Use Committee of Kindai University and were conducted according to the Kindai University Animal Experimentation Regulations (approval number KAAG-31–008). All efforts were made to minimize their suffering. Animal experiments were conducted using mice. During the acclimation period, 4-week-old male ddY mice (Japan SLC, Inc., Shizuoka, Japan) were provided free access to a commercial diet (MF; Oriental Yeast Co., Ltd., Tokyo, Japan) and water. They were maintained at 25 ± 1 °C on a standard 12-h/12-h light/dark cycle. The study was carried out in compliance with the ARRIVE guidelines.

### Evaluation of the distribution of turmerones in mice

After 5 days of acclimation, the mice were divided into two groups: Oa-T group, no inhalation + turmeric solution (0.04 mg/g); and I-T group, volatile turmeric oil inhalation (60 min). The solution was orally administered to mice in the Oa-T groups. Oral administration was by gavage. Turmeric oil contained in the turmeric solution used in the Oa-T group was emulsified with 2% CMC. After treatment, the mice in the Oa-T group were kept in normal air for 30 min. Mice in the I-T group were inhaled into turmeric oil for 60 min. For the preparation, 10 mL of turmeric oil was soaked in a cotton, placed in a net, and hung in a 5000-mL flask using a paper string and silicone stopper. The flask was closed with a silicone stopper and filled with volatile turmerones. After 10 min, the mice in the I-T group were placed in the flask and left for 60 min with the stopper closed during the inhalation (Supplementary Fig. S1a). All mice were euthanized before dissection using sodium pentobarbital over-anesthetization, and blood (1 mL) was obtained from the inferior vena cava during anesthetization. Turmerones in serum or organs were measured using an ultra-performance liquid chromatography–mass spectrometry (UPLC-MS) system (LC–MS-8050; Shimadzu Co., Ltd., equipped with Lab Solutions ver. 5.75). The quantitative conditions for turmerones in serum or organs was described in a previous study^18^.

### Evaluation of the effect of turmeric oil inhalation on adipose tissue

After 5 days of acclimation, mice were divided into four groups: the C group, control diet + no inhalation; the CT group, control diet + volatile turmeric oil inhalation (60 min); the HF group, high-fat diet + no inhalation; and the HFT group, high-fat diet + volatile turmeric oil inhalation (60 min). The experimental period was set at 50 days. The mice were provided free access to water and each diet. The diet compositions are shown in Table 1. Mice in the inhalation groups were inhaled into volatile turmerones for 60 min (3 times/week; Monday, Wednesday, and Friday (Supplementary Fig. S1b)). Blood glucose levels were evaluated the day before dissection. Blood was collected from the tail vein after 3 h of fasting. On the day of measurement, all mice were euthanized using sodium pentobarbital over-anesthetization, and blood (1 mL) was obtained from the inferior vena cava. The left cardiac ventricle of each mouse was subsequently perfused with an isotonic sodium chloride solution. The epididymal fat was isolated and weighed. It was then soaked in 4% paraformaldehyde (PFA), fixed overnight at 4 °C, and embedded in paraffin.

**Table 1:** Diet composition.

	Control diet (g)	High-fat diet (g)
Choline chloride	2	2
Cystine	3	3
ANI-93 vitamin mix	10	10
ANI-93 mineral mix	32	32
Cellulose	50	50
Sucrose	68.8	68.8
Casein	200	200
Cornswatch	506.2	–
Maltodextrin	125	125
Calcium hydrogen Phosphate dihydrate	16.4	16.4
Soyabean oil	25	25
Lard	20	245
Water	500	30
Total Kcal/g	1058.4 3.85	777.2 5.24

### Serum parameters

Blood obtained from the tail vein on the day before dissection (day 49) was measured for blood glucose level using a GLUCOCARD Plus Care GT-1840 (ARKRAY, Kyoto, Japan) and G sensor (ARKRAY). The blood obtained from the inferior vena cava was centrifuged at 4 °C for 10 min at 3000 g to obtain serum. Serum levels of triglycerides (FUJIFILM Wako Pure Chemical Co., Osaka, Japan), total cholesterol (FUJIFILM Wako Pure Chemical Co.), and insulin (Morinaga Institute of Biological Science, Inc., Kanagawa, Japan) were measured using a commercially available kit.

### Histological analysis

For histopathological analysis, epididymal fat collected from animal experiments was soaked in 4% paraformaldehyde (PFA), fixed overnight at 4 °C, and then embedded in paraffin. Subsequently, 4-μm-thick sections were obtained using a rotary microtome. Epididymal fat was stained with hematoxylin and eosin. Quantitative analysis of histological staining was performed using ImageJ software (National Institutes of Health, Bethesda, MD, USA).

### Statistical analyses

Statistical differences were determined using the Mann–Whitney U test (for two groups) or Tukey–Kramer test (for multiple groups), and p < 0.05 was considered statistically significant. Statistical analyses were performed using the Stat View 5.0 software (SAS Institute, Cary, USA).

## Supplementary Information

Supplementary Information.

## Data Availability

All data generated or analysed during this study are included in this published article and its supplementary information files.
