# Supplementary material for: Inhaled turmerones can be incorporated in the organs via pathways different from oral administration and can affect weight-gain of mice
Source: Sci Rep. 2022 Jun 30;12:11039. doi: 10.1038/s41598-022-15168-9 (PMC9247068; doi:10.1038/s41598-022-15168-9)
Supplement: Supplementary file 1 — Supplementary Information. [file 41598_2022_15168_MOESM1_ESM.pdf]

Supplementary Figure S1

(a) Experimental scheme (Oa-T, E-T)

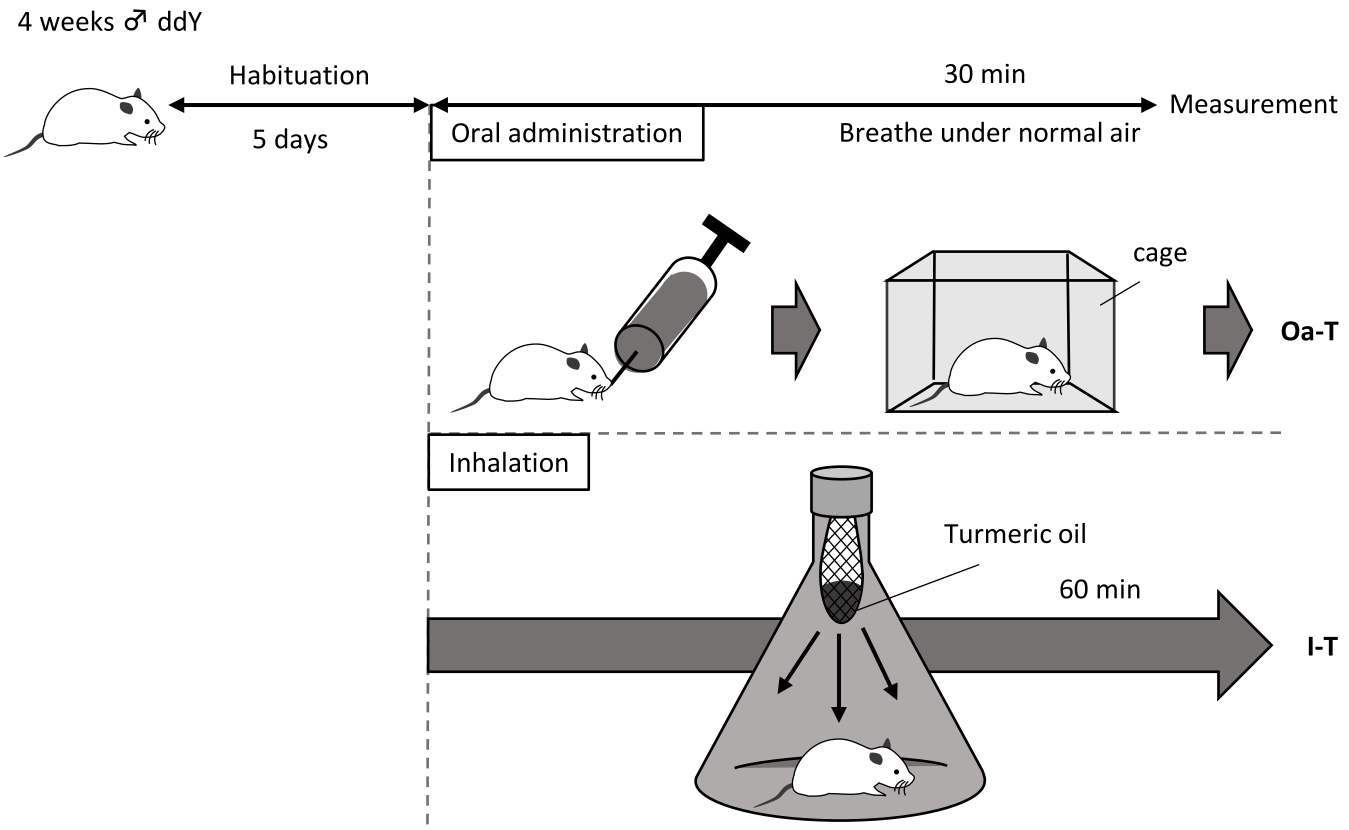

(b) Experimental scheme (C, CT, HF, HFT)

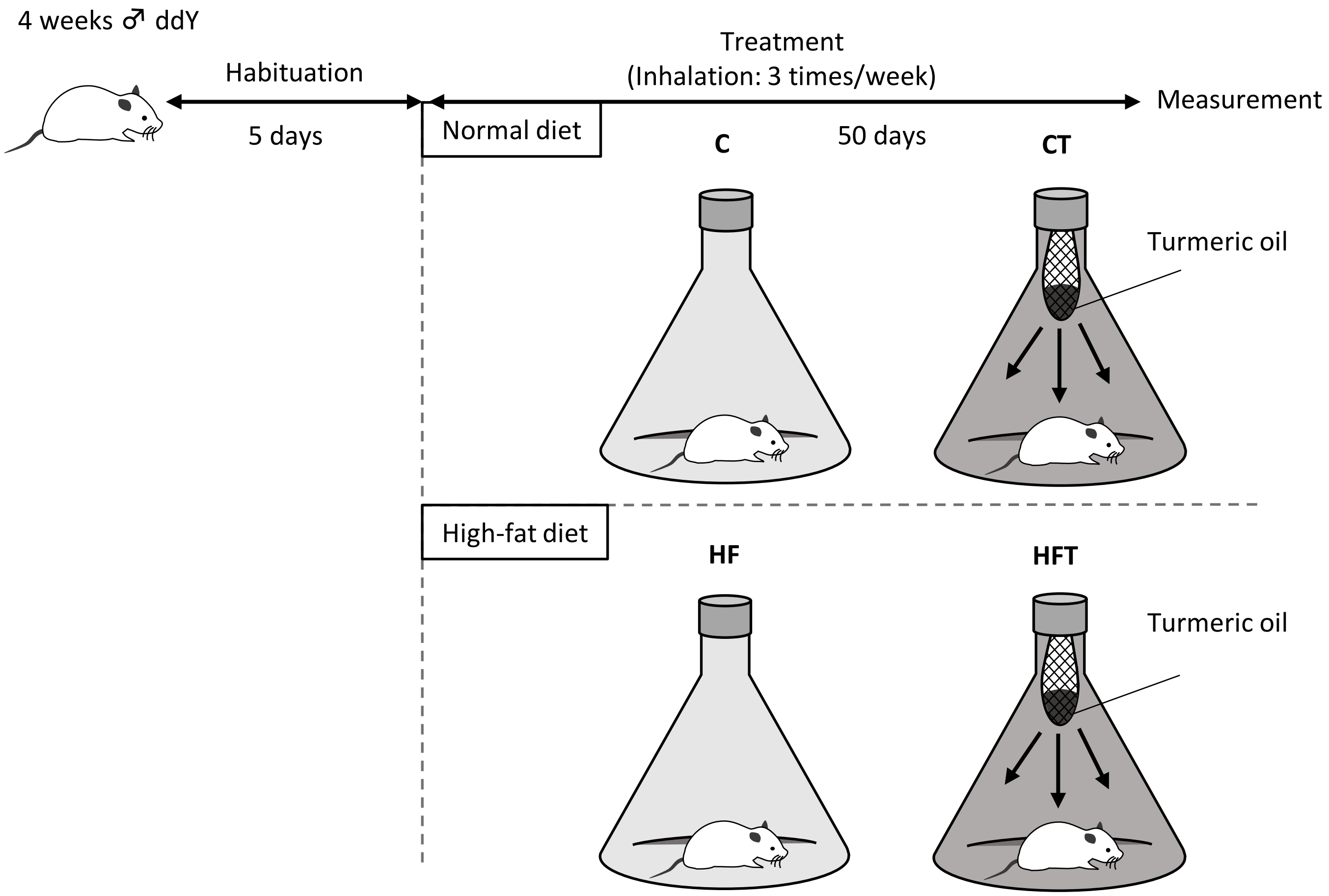

Supplementary Figure S1 Experimental scheme.

(a) Evaluation of transfer ratio of turmerones between oral administration and inhalation.

(b) Evaluation of the effect of turmeric oil inhalation on adipose tissue.
